# Multicenter clinical evaluation of a fully automated multiplex HSV-1, HSV-2, and VZV real-time PCR assay

**DOI:** 10.1128/jcm.00535-26

**Published:** 2026-08-14

**Authors:** Joseph Yao, Erin McElvania, Mohammad Al-Ghoul, Parul Patel, Rupinder Kular, Jeffrey Wuitschick, Shihai Huang, John Birdsall, Ekaterina Pestova, Neelam Dhiman

**Affiliations:** 1 Mayo Clinic Laboratories 682343, Rochester, Minnesota, USA; 2 Department of Pathology and Laboratory Medicine, Endeavor Health 3271, Evanston, Illinois, USA; 3 Department of Pathology, University of Chicago, Pritzker School of Medicine 2462, Chicago, Illinois, USA; 4 LabCorp, San Antonio, Texas, USA; 5 Molecular Diagnostics at Abbott, Des Plaines, Illinois, USA; 6 Quest Diagnostics Inc., Lewisville, Texas, USA; St Jude Children's Research Hospital, Memphis, Tennessee, USA

**Keywords:** Multiplex real-time PCR, HSV, VZV, high-throughput assay, viral detection and differentiation

## Abstract

Herpes simplex virus type 1 (HSV-1), herpes simplex virus type 2 (HSV-2), and varicella-zoster virus (VZV) are associated with a range of clinical manifestations depending on the anatomical site of infection. HSV and VZV lesions often exhibit similar phenotypical characteristics, making clinical differentiation challenging. Detection and differentiation of HSV-1, HSV-2, and VZV are essential for diagnosis and treatment management. An automated multiplex real-time PCR assay, Alinity m HSV 1 & 2/VZV assay, was developed to detect and differentiate HSV-1, HSV-2, and VZV. The assay was validated for use with cutaneous and mucocutaneous lesion swab specimens collected in commercial viral transport media from symptomatic patients. The assay demonstrated a limit of detection of 5.90 TCID_50_/mL for HSV-1, 2.07 TCID_50_/mL for HSV-2, and 0.055 TCID_50_/mL for VZV. Testing with clinical swab specimens demonstrated greater than 95% clinical sensitivity and specificity for all three analytes. The Alinity m HSV 1 & 2/VZV assay provides a reliable and accurate method for detection and differentiation of HSV-1, HSV-2, and VZV in lesion swab specimens for improved diagnostic accuracy and patient management.

**IMPORTANCE:**

Herpes simplex virus type 1 (HSV-1), herpes simplex virus type 2 (HSV-2), and varicella-zoster virus (VZV) can cause skin and mucocutaneous lesions that often appear clinically similar, making accurate diagnosis difficult without laboratory testing. Rapid and reliable identification of the causative virus is important because patient counseling, treatment decisions, and infection management differ among these infections. In this multicenter study, a fully automated molecular assay was evaluated that simultaneously detects and differentiates HSV-1, HSV-2, and VZV from a single-lesion swab specimen. Testing of more than 1,200 clinical specimens demonstrated high sensitivity and specificity for all three viruses. The assay also offers a streamlined workflow with minimal hands-on time and rapid turnaround. These findings support the use of this multiplex assay as a practical diagnostic tool that can improve laboratory efficiency and provide clinicians with timely, accurate results to guide patient care.

## INTRODUCTION

Human herpesviruses belong to the *Herpesviridae* family of DNA viruses and are divided into alpha, beta, and gamma subfamilies. *Alphaherpesvirinae* includes HSV-1, HSV-2, and VZV that have a genome size ranging from 125 to 240 kbp containing about 70 to 165 genes (1, 2).

HSV-1 and HSV-2 share a similar genome with approximately 80% homology of their protein-coding regions. These viruses are closely related and cause a variety of illnesses depending on the anatomical site where the infection initiates, the immune state of the host, and whether the infection is primary or recurrent. HSV is transmitted by skin-to-skin contact or by contact with bodily fluids. Viral particles shed from mucocutaneous lesions or from asymptomatic shedding of an infected person, enter epithelial cells of a new host through breaks in the skin or mucosa, and begin replicating. Typically, HSV-1 is transmitted through oral-to-oral contact, and HSV-2 is sexually transmitted. Primary infection at the dermal-epidermal junction produces characteristic skin lesions that begin as macules and papules, culminating in vesicles containing copious amounts of infectious virus. These vesicles form pustules that rupture in approximately 2 days; the resulting ulcers and crusts typically form within 4 days. The HSV DNA can be detected throughout all visible stages of the infection, including the crust. Following a primary infection, the virus may become latent in local sensory ganglia that may be periodically reactivated to cause symptomatic lesions (2–4).

VZV infections are extremely contagious and occur through inhalation of infectious droplets or through contact with skin lesions. Clinically, the virus has two manifestations causing varicella, also known as chickenpox, and zoster, also known as shingles. VZV infection begins with virus replication in epithelial cells of the upper respiratory mucosa. The virus predominantly infects T cells, resulting in viremia, causing asymptomatic infection of many organs, and eventually reaches the skin. Approximately 2 weeks after primary infection, a rash appears, which progresses to papules and then vesicles, eventually to crusting. During primary VZV infection, the virus accesses sensory nerve cell bodies in ganglia, resulting in latent infection for life. In case of reactivation, VZV travels along the sensory nerve until it reaches the skin and causes the symptoms of zoster, which is characterized by severe pain and burning followed by a painful vesicular rash usually limited to the dermatomes (1, 5–10).

HSV and VZV lesions can be indistinguishable phenotypically; thus, clinical diagnosis of herpes and varicella/zoster can be challenging without diagnostic testing. Additionally, type-specific diagnosis of HSV is recommended as differences between HSV-1 and HSV-2 infections can impact the recurrence rate, sub-clinical viral shedding, and transmission risk (4, 11). The most common method of HSV-1, HSV-2, and VZV diagnostic testing involves swabbing the lesions of a symptomatic patient followed by nucleic acid amplification testing (NAAT). NAAT testing has previously been shown to provide accurate detection of HSV-1, HSV-2, and VZV with high sensitivity and specificity (12).

In this study, we evaluated the analytical and clinical performance of the real-time PCR-based Alinity m HSV 1 & 2/VZV assay (Molecular Diagnostics at Abbott, Des Plaines, IL) for detection and differentiation of HSV-1, HSV-2, and VZV from cutaneous and mucocutaneous lesions. Alinity m HSV 1 & 2/VZV testing was performed using the automated Alinity m System (Molecular Diagnostics at Abbott), a continuous and random-access molecular analyzer with a time to first result of less than 2 h. Accurate detection of HSV-1, HSV-2, and VZV in conjunction with clinical presentation provides clinicians the information they need for appropriate patient management (8).

## MATERIALS AND METHODS

### Clinical specimens

Clinical swab specimens (*n* = 1,291) from 1,064 symptomatic individuals with skin lesions suspected of HSV-1, HSV-2, and/or VZV infection were included in the study (one to five lesion swab specimens per subject). When multiple specimens were collected from a single subject, independent lesion swab samples were obtained from separate lesions. Of these, 1,259 specimens (1,044 subjects) were included in the analysis for at least one of the analytes (1,257, 1,258, and 1,255 for HSV-1, HSV-2, and VZV, respectively). The specimens included 642 cutaneous lesions, 614 mucocutaneous lesions, and three uncategorized lesions. The study population included pediatric (<18 years) and adult participants, with subjects ranging from <1 year to >90 years in age. Pediatric subjects represented 10% of the total population. The cohort comprised 606 females and 436 males, which included one male-to-female transgender individual and two subjects whose gender was unknown. Specimens were collected from US collection sites or obtained from commercial vendors (iProcess Global Research Inc., Irving, TX). The specimens were collected in BD-UVT (*n* = 344), Copan-UTM (*n* = 506), and Remel-M4RT (*n* = 409) media and tested fresh (without freezing) or stored frozen at ≤−70°C until testing was performed. All specimens were deidentified and were obtained under an Independent Ethics Committee (IEC) or Institutional Review Board (IRB)-approved protocol with informed consent or leftover, not individually identifiable specimens obtained according to applicable laws and guidance regarding informed consent and the use of leftover human specimens.

### Assay platform

Specimen testing was performed using the Alinity m HSV 1 & 2/VZV assay that is run on the Alinity m instrument. Lesion swab specimens collected in viral transport medium (e.g., Copan UTM, BD UVT, and Remel M4RT) were placed on the sample racks on the Alinity m system. A sample volume of 0.45 mL was processed for nucleic acid extraction using magnetic microparticle technology to facilitate nucleic acid capture, wash, and elution. The purified viral nucleic acids were then combined with the PCR amplification reagents containing primers and probes for HSV-1, HSV-2, and VZV. An exogenous internal control and its associated primers and probe were also included in the amplification reagent to demonstrate proper sample processing and evaluate potential PCR inhibitors in the samples. PCR amplification and real-time fluorescence detection were performed by the Alinity m system to generate cycle numbers when the fluorescent signal is detected for each analyte.

### Clinical study

All specimens were tested with the Alinity m HSV 1 & 2/VZV assay along with two comparator NAATs for each analyte. Testing with the Alinity m assay and comparator assays was performed independently at participating laboratories. Operators performing testing were blinded to the results of the other assays to the extent feasible within site workflows. For HSV-1 and HSV-2, the comparator assays were the Simplexa HSV 1 & 2 Direct assay (DiaSorin Molecular LLC, Cypress, CA) and an HSV Laboratory Developed Test (HSV LDT; Quest Diagnostics Inc., Lewisville, TX) for real-time PCR-based detection and differentiation of HSV-1 and HSV-2. For VZV, the comparator assays were the Simplexa VZV Swab Direct assay (DiaSorin Molecular LLC) performed on the LIAISON MDX instrument and a VZV LDT (Quest Diagnostics Inc.) for real-time PCR-based detection of VZV. A composite comparator (CC) was established for each analyte. If either or both NAATs were positive, the CC would be positive; if both comparator NAATs were negative, the CC would be negative. Data analysis was conducted using predefined statistical methods established prior to data unblinding. Clinical sensitivity and specificity were calculated for each analyte based on true positive or true negative results, as defined by CC. Confidence intervals were calculated using Wilson’s score method.

### Analytical sensitivity

Limit of detection (LoD) for HSV-1, HSV-2, and VZV was determined by testing seven panel members (PMs) that were prepared by diluting HSV-1-, HSV-2-, or VZV-cultured virus into the negative clinical matrix. HSV-1- (catalog number VR-539, lot 63953722), HSV-2- (catalog number VR-540, lot 70032981), and VZV-cultured (catalog number VR-1367, lot 70033832) viruses were obtained from American Type Culture Collection (ATCC). The negative clinical matrix was prepared by pooling negative clinical lesion swab specimens obtained from commercial vendors. The concentration of the virus in the PMs ranged from 0.7 to 10.0 TCID_50_/mL for HSV-1, 0.30 to 7.0 TCID_50_/mL for HSV-2, and 0.004 to 0.016 TCID_50_/mL for VZV. For each PM, eight replicates were tested per day for 3 days across three amplification reagent lots and three Alinity m instruments, with each lot of reagents assigned to a specific instrument.

### Assay reproducibility performance

Assay reproducibility was evaluated by testing four PMs that were targeted to four different levels (positive, low positive, high negative, and negative). The PMs were prepared by spiking HSV-1-, HSV-2-, and VZV-cultured viral particles into negative matrix to achieve the targeted level at 5× LoD (positive), 1–2× LoD (low positive), and <1 × LoD (high negative). The testing was performed at the following three laboratories using a total of three amplification reagent lots: Quest Diagnostics Inc. (Lewisville, TX), Mayo Clinic Laboratories (Rochester, MN), and LabCorp (San Antonio, TX). Each laboratory site tested six replicates of each PM using two amplification reagent lots, on 5 days. The PROC MIXED procedure with the MIVQUE0 option in SAS was used to produce variance components. The SD and %CV were estimated for the within-run component, the between-run component, and the between-lot component for each panel member. The total assay variability was defined as the sum of the within-run/day (residual error) component, the between-run/day component, and the between-lot component estimates of variability.

## RESULTS

### Clinical performance

For HSV-1, 127 specimens were positive with both Alinity assay and CC, and 1,117 were negative with both Alinity assay and CC. HSV-1 clinical sensitivity was 96.2% (127/132) and specificity was 99.3% (1,117/1,125) (Table 1). Out of the five specimens that were positive by CC and negative by Alinity m assay, three specimens had no amplification with the Alinity m assay and two specimens demonstrated PCR amplification with cycle number (CN) values of 32.62 and 32.16, which were beyond the assay cutoff of 31.00 CN for positivity. All eight specimens that were positive with the Alinity assay but were negative per CC results had CN values near the assay cutoff, suggesting that the low-level target was detected by Alinity assay that was not detected by either one of the comparator assays (Table 2).

**TABLE 1:** Clinical performance for HSV-1, HSV-2, and VZV across lesion swab specimens^*a*^

Analyte	Lesion type	Total N	CC +Alinity +	CC +Alinity −	CC − Alinity −	CC − Alinity +	Estimate sensitivity (%) (95% CI)	Estimate specificity (%) (95% CI)
HSV-1	Cutaneous	641	38	1	599	3	97.4 (86.8, 99.5)	99.5 (98.5, 99.8)
	Mucocutaneous	613	89	3	516	5	96.7 (90.8, 98.9)	99.0 (97.8, 99.6)
	Uncategorized	3	0	1	2	0	0.0 (0.0, 79.3)	100.0 (34.2, 100.0)
	Total	1,257	127	5	1,117	8	96.2 (91.4, 98.4)	99.3 (98.6, 99.6)
HSV-2	Cutaneous	641	63	2	574	2	96.9 (89.5, 99.2)	99.7 (98.7, 99.9)
	Mucocutaneous	614	63	0	548	3	100.0 (94.3, 100.0)	99.5 (98.4, 99.8)
	Uncategorized	3	1	0	2	0	100.0 (20.7, 100.0)	100.0 (34.2, 100.0)
	Total	1,258	127	2	1,124	5	98.4 (94.5, 99.6)	99.6 (99.0, 99.8)
VZV	Cutaneous	640	40	0	600	0	100.0 (91.2, 100.0)	100.0 (99.4, 100.0)
	Mucocutaneous	612	5	2	605	0	71.4 (35.9, 91.8)	100.0 (99.4, 100.0)
	Uncategorized	3	0	0	3	0	N/A^*b*^	100.0 (43.9, 100.0)
	Total	1,255	45	2	1,208	0	95.7 (85.8, 98.8)	100.0 (99.7, 100.0)

^*a*^ The table summarizes total specimens tested, results (CC and Alinity-positive/-negative combinations), and corresponding estimates of sensitivity and specificity with 95% confidence intervals. “Uncategorized” refers to specimens without an assigned lesion type.

^*b*^ “N/A” indicates that the sensitivity could not be estimated due to the absence of positive cases in that subgroup.

**TABLE 2:** Results of discordant samples between the composite comparator (CC) and Alinity m results for HSV-1^*a*^

#	HSV-1 discordant result	NAAT1 (Simplexa) HSV-1 result	NAAT2 (Quest) HSV-1 result	Composite comparator HSV-1 result	Alinity m HSV-1 result	Alinity m HSV-1 CN value^*b*^
1	Alinity m +, CC−	Not detected	Not detected	Negative	HSV-1-positive	29.41
2	Alinity m +, CC−	Not detected	Not detected	Negative	HSV-1-positive	30.57
3	Alinity m +, CC−	Not detected	Not detected	Negative	HSV-1-positive	30.72
4	Alinity m +, CC−	Not detected	Not detected	Negative	HSV-1-positive	30.13
5	Alinity m +, CC−	Not detected	Not detected	Negative	HSV-1-positive	30.74
6	Alinity m +, CC−	Not detected	Not detected	Negative	HSV-1-positive	28.37
7	Alinity m +, CC−	Not detected	Not detected	Negative	HSV-1-positive	30.25
8	Alinity m +, CC−	Not detected	Not detected	Negative	HSV-1-positive	29.90
9	Alinity m −, CC+	Not detected	Detected	Positive	HSV-1-negative	NA
10	Alinity m −, CC+	Detected	Not detected	Positive	HSV-1-negative	32.62
11	Alinity m −, CC+	Detected	Not detected	Positive	HSV-1-negative	32.16
12	Alinity m −, CC+	Not detected	Detected	Positive	HSV-1-negative	NA
13	Alinity m −, CC+	Detected	Not detected	Positive	HSV-1-negative	NA

^*a*^ Alinity m HSV-1 CN values are shown for positive detections; “NA” indicates no CN value was generated. A composite comparator (CC) was established for each analyte. The CC result was positive, if either or both NAATs were positive. The CC result was negative, if both comparator NAATs were negative.

^*b*^ Excludes five unread cycles.

For HSV-2, 127 specimens were positive with both Alinity assay and CC, and 1,124 were negative with both Alinity assay and CC. HSV-2 clinical sensitivity was 98.4% (127/129) and specificity was 99.6% (1,124/1,129) (Table 1). Out of the two specimens that were positive by CC and negative by Alinity assay, one specimen had no amplification with the Alinity m assay, while one specimen demonstrated PCR amplification with a CN value of 33.57, which was beyond the assay cutoff of 33.00 CN for positivity. All five specimens that were positive with Alinity m assay, but were negative per CC, had CN values near the positivity cutoff, suggesting that the low-level target was detected by Alinity assay that was not detected by either one of the comparator assays (Table 3).

**TABLE 3:** Results of discordant samples between the composite comparator (CC) and Alinity m results for HSV-2^*a*^

#	HSV-2 discordant result	NAAT1 (Simplexa) HSV-2 result	NAAT2 (Quest) HSV-2 result	Composite comparator HSV-2 result	Alinity m HSV-2 result	Alinity m HSV-2 CN value^*b*^
1	Alinity m +, CC−	Not detected	Not detected	Negative	HSV-2-positive	32.67
2	Alinity m +, CC−	Not detected	Not detected	Negative	HSV-2-positive	31.11
3	Alinity m +, CC−	Not detected	Not detected	Negative	HSV-2-positive	31.27
4	Alinity m +, CC−	Not detected	Not detected	Negative	HSV-2-positive	31.17
5	Alinity m +, CC−	Not detected	Not detected	Negative	HSV-2-positive	31.71
6	Alinity m −, CC+	Detected	Not detected	Positive	HSV-2-negative	33.57
7	Alinity m −, CC+	Not detected	Detected	Positive	HSV-2-negative	NA

^*a*^ Alinity m HSV-2 CN values are shown for positive detections; “NA” indicates no CN value was generated. A composite comparator (CC) was established for each analyte. The CC result was positive, if either or both NAATs were positive. The CC result was negative, if both comparator NAATs were negative.

^*b*^ Excludes five unread cycles.

For VZV, 45 specimens were positive with both Alinity assay and CC, and 1,208 were negative with both Alinity assay and CC. VZV clinical sensitivity was 95.7% (45/47) and specificity was 100.0% (1,208/1,208) (Table 1). Out of the two specimens that were positive by CC and negative by Alinity assay, one specimen had no amplification with the Alinity assay, and one specimen demonstrated PCR amplification with a CN value of 33.38, which was beyond the assay cutoff of 33.00 CN for positivity (Table 4).

**TABLE 4:** Results of discordant samples between the composite comparator (CC) and Alinity m results for VZV^*a*^

#	VZV discordant result	NAAT1 (Simplexa) VZV result	NAAT2 (Quest) VZV result	Composite comparator VZV result	Alinity m VZV result	Alinity m VZV CN value^*b*^
1	Alinity m −, CC+	Detected	Not detected	Positive	VZV-negative	33.38
2	Alinity m −, CC+	Not detected	Detected	Positive	VZV-negative	NA

^*a*^ Alinity m VZV CN values are shown for positive detections; “NA” indicates no CN value was generated. A composite comparator (CC) was established for each analyte. The CC result was positive, if either or both NAATs were positive. The CC result was negative, if both comparator NAATs were negative.

^*b*^ Excludes five unread cycles.

**Fig 1:**
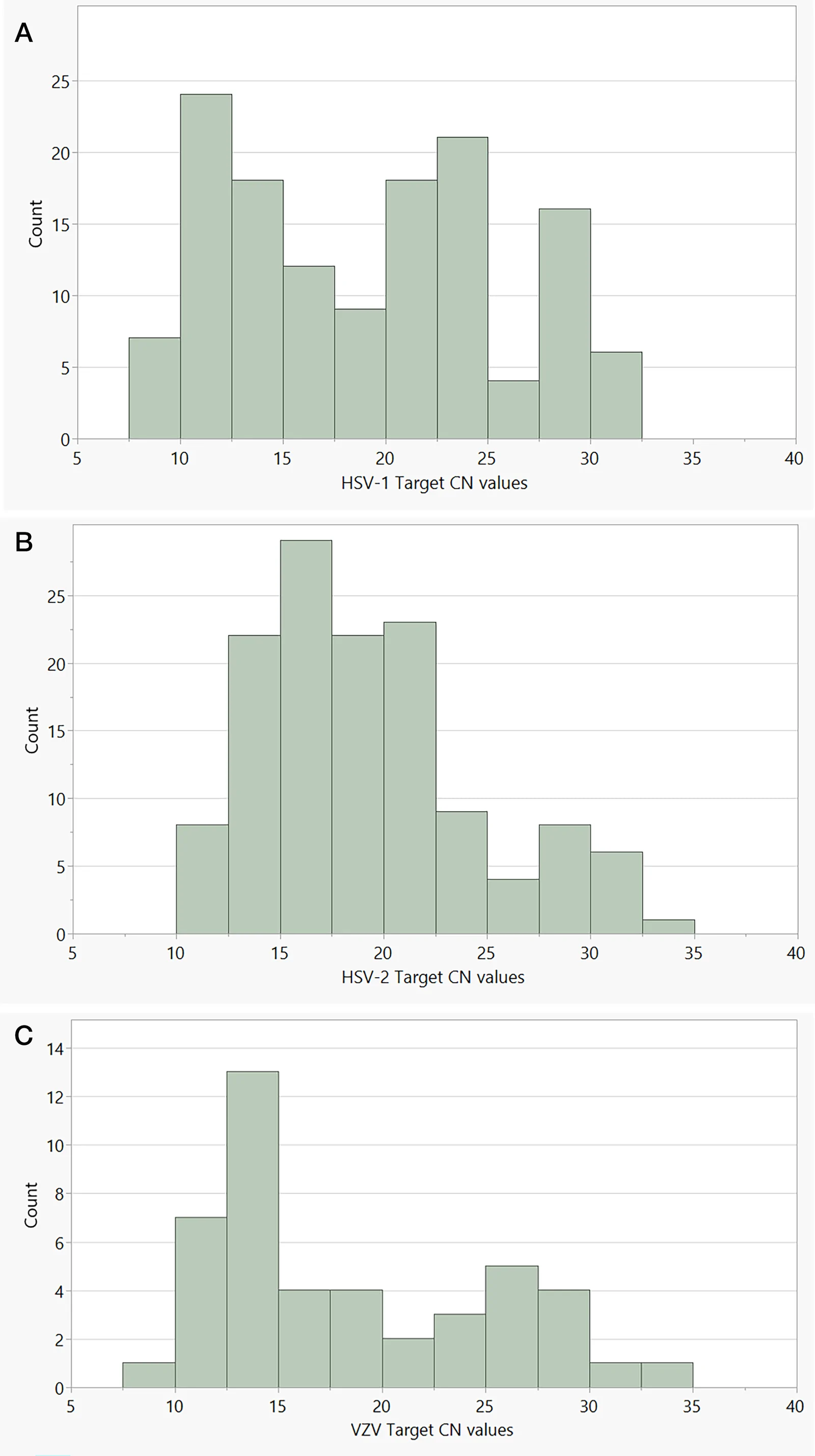
Distribution of HSV-1 (**A**), HSV-2 (**B**), and VZV (**C**) target cycle number (CN) values among positive Alinity m results. The histogram illustrates the frequency and range of observed CN values (excludes five unread cycles) across the tested specimens.

The cycle number distribution from all Alinity m HSV-1-, HSV-2-, and VZV-positive results is shown in Fig. 1.

The specimens used in the testing included fresh and frozen specimens, and both conditions demonstrated comparable performance. Clinical sensitivity and specificity for all three analytes for both fresh and frozen samples are shown in Table 5.

**TABLE 5:** Clinical performance of the Alinity m assay for HSV-1, HSV-2, and VZV under fresh and frozen storage conditions^*a*^

Analyte	Storage condition	Total N	CC + Alinity +	CC + Alinity −	CC − Alinity −	CC − Alinity +	Estimate sensitivity (%) (95% CI)	Estimate specificity (%) (95% CI)
HSV-1	Fresh	1,017	93	3	913	8	96.9 (91.2, 98.9)	99.1 (98.3, 99.6)
HSV-2	Fresh	1,018	79	1	936	2	98.8 (93.3, 99.8)	99.8 (99.2, 99.9)
VZV	Fresh	1,017	36	2	979	0	94.7 (82.7, 98.5)	100.0 (99.6, 100.0)
HSV-1	Frozen	240	34	2	204	0	94.4 (81.9, 98.5)	100.0 (98.2, 100.0)
HSV-2	Frozen	240	48	1	188	3	98.0 (89.3, 99.6)	98.4 (95.5, 99.5)
VZV	Frozen	238	9	0	229	0	100.0 (70.1, 100.0)	100.0 (98.4, 100.0)

^*a*^ The table summarizes specimen counts, results with CC, and estimated sensitivity and specificity with 95% confidence intervals.

### Analytical sensitivity

The limit of detection (LoD) for HSV-1, HSV-2, and VZV was determined by Probit analysis. The analysis determined that the concentration detected with 95% probability for HSV-1, HSV-2, and VZV was 5.90 TCID_50_/mL, 2.07 TCID_50_/mL, and 0.055 TCID_50_/mL, respectively (Table 6). The detection rate for each PM and for all three analytes is listed in Table 7. A competitive interference study demonstrated that 100% of replicates for HSV-1, HSV-2, or VZV targeted at 3× LOD were detected in the presence of high concentrations (≥1,000 × LoD) of the other on-panel analytes (data not shown).

**TABLE 6:** Analytical sensitivity of the Alinity m assay for HSV-1, HSV-2, and VZV^*a*^

Analyte	Strain	ATCC catalog number	LoD (TCID_50_/mL)
HSV-1	MacIntyre	VR-539	5.90
HSV-2	MS	VR-540	2.07
VZV	Ellen	VR-1367	0.055

^*a*^ The reference strains obtained from ATCC were used to determine the limit of detection (LoD) for HSV-1, HSV-2, and VZV. Reported values (TCID₅₀/mL) reflect the lowest viral concentrations consistently detected for each strain.

**TABLE 7:** Detection rates for HSV-1, HSV-2, and VZV across a range of low viral concentrations^*a*^

Analyte	Target concentration (TCID_50_/mL)	Total number of replicates	Total number of replicates detected	Detection rate (%)
HSV-1	10	70	70	100.0%
	3	72	66	91.7%
	2	72	51	70.8%
	1	71	37	52.1%
	0.9	72	33	45.8%
	0.8	70	29	41.4%
	0.7	72	26	36.1%
HSV-2	7	70	70	100.0%
	2	72	71	98.6%
	0.55	72	39	54.2%
	0.50	71	50	70.4%
	0.40	72	43	59.7%
	0.35	70	35	50.0%
	0.30	72	26	36.1%
VZV	0.0160	70	69	98.6%
	0.0100	72	41	56.9%
	0.0040	72	8	11.1%
	0.0032	71	24	33.8%
	0.0024	72	10	13.9%
	0.0020	70	11	15.7%
	0.0016	72	13	18.1%

^*a*^ The table presents the number of replicates tested at each TCID₅₀/mL level, the number detected, and the resulting detection rates.

### Assay reproducibility performance

To evaluate assay reproducibility, four panel members were tested at three testing sites. All replicates (100%) of 5× LoD for all three sites were detected for all three analytes. The rate of detection for 1–2× LoD PM was 98.9% for HSV-1 and 100% for HSV-2 and VZV across all sites combined. All replicates of negative PM were negative across all three sites. Mean CN values, standard deviation, and %CV for all PMs at different sites are listed in Table 8.

**TABLE 8:** Precision performance of the Alinity m assay for HSV-1, HSV-2, and VZV across three testing sites using panel members spanning 5× LOD, 1–2× LOD, and sub-LOD concentrations^*a*^

Analyte	Testing Site	Panel member	Positivity rate	Positivity rate (95% CI)	Average CN^*b*^	Within-run/day		Between-run/day		Between-lot		Total	
						SD	% CV	SD	% CV	SD	% CV	SD	% CV
HSV-1	1	5× LoD	60/60	100.00% (94.0%, 100.0%)	27.15	0.305	1.1	0.061	0.2	0.148	0.5	0.345	1.3
		1×–2× LoD	59/60	98.30% (91.1%, 99.7%)	28.86	0.351	1.2	0.047	0.2	0.100	0.3	0.368	1.3
		Sub-LoD (<1 × LoD)	41/60	68.30% (55.8%, 78.7%)	30.48	0.402	1.3	0.045	0.1	0.000	0.0	0.404	1.3
	2	5× LoD	60/60	100.00% (94.0%, 100.0%)	27.31	0.322	1.2	0.130	0.5	0.000	0.0	0.347	1.3
		1×–2× LoD	60/60	100.00% (94.0%, 100.0%)	29.03	0.439	1.5	0.000	0.0	0.000	0.0	0.439	1.5
		Sub-LoD (<1 × LoD)	23/60	38.30% (27.1%, 51.0%)	30.59	0.400	1.3	0.000	0.0	0.000	0.0	0.400	1.3
	3	5× LoD	60/60	100.00% (94.0%, 100.0%)	27.17	0.338	1.2	0.195	0.7	0.114	0.4	0.406	1.5
		1×–2× LoD	59/60	98.30% (91.1%, 99.7%)	28.98	0.443	1.5	0.000	0.0	0.000	0.0	0.443	1.5
		Sub-LoD (<1 × LoD)	25/60	41.70% (30.1%, 54.3%)	30.54	0.417	1.4	0.000	0.0	0.000	0.0	0.417	1.4
HSV-2	1	5× LoD	60/60	100.00% (94.0%, 100.0%)	29.44	0.275	0.9	0.136	0.5	0.000	0.0	0.307	1.0
		1×–2× LoD	60/60	100.00% (94.0%, 100.0%)	30.37	0.331	1.1	0.172	0.6	0.188	0.6	0.418	1.4
		Sub-LoD (<1 × LoD)	29/60	48.30% (36.2%, 60.7%)	32.41	0.442	1.4	0.000	0.0	0.143	0.4	0.465	1.4
	2	5× LoD	60/60	100.00% (94.0%, 100.0%)	29.67	0.365	1.2	0.133	0.4	0.000	0.0	0.389	1.3
		1×–2× LoD	60/60	100.00% (94.0%, 100.0%)	30.70	0.425	1.4	0.000	0.0	0.000	0.0	0.425	1.4
		Sub-LoD (<1 × LoD)	16/60	26.70% (17.1%, 39.0%)	32.46	0.374	1.2	0.000	0.0	0.174	0.5	0.413	1.3
	3	5× LoD	60/60	100.00% (94.0%, 100.0%)	29.47	0.284	1.0	0.156	0.5	0.130	0.4	0.349	1.2
		1×–2× LoD	60/60	100.00% (94.0%, 100.0%)	30.61	0.411	1.3	0.130	0.4	0.280	0.9	0.514	1.7
		Sub-LoD (<1 × LoD)	19/60	31.70% (21.3%, 44.2%)	32.25	0.414	1.3	0.138	0.4	0.000	0.0	0.436	1.4
VZV	1	5× LoD	60/60	100.00% (94.0%, 100.0%)	27.25	0.577	2.1	0.142	0.5	0.082	0.3	0.600	2.2
		1×–2× LoD	60/60	100.00% (94.0%, 100.0%)	29.08	0.345	1.2	0.215	0.7	0.144	0.5	0.432	1.5
		Sub-LoD (<1 × LoD)	38/60	63.30% (50.7%, 74.4%)	32.25	0.517	1.6	0.000	0.0	0.102	0.3	0.527	1.6
	2	5× LoD	60/60	100.00% (94.0%, 100.0%)	27.60	0.303	1.1	0.156	0.6	0.000	0.0	0.341	1.2
		1×–2× LoD	60/60	100.00% (94.0%, 100.0%)	29.39	0.561	1.9	0.063	0.2	0.000	0.0	0.565	1.9
		Sub-LoD (<1 × LoD)	32/60	53.30% (40.9%, 65.4%)	32.05	0.686	2.1	0.000	0.0	0.000	0.0	0.686	2.1
	3	5× LoD	60/60	100.00% (94.0%, 100.0%)	27.28	0.278	1.0	0.165	0.6	0.000	0.0	0.323	1.2
		1×–2× LoD	60/60	100.00% (94.0%, 100.0%)	29.19	0.377	1.3	0.137	0.5	0.000	0.0	0.401	1.4
		Sub-LoD (<1 × LoD)	42/60	70.00% (57.5%, 80.1%)	32.29	0.532	1.6	0.000	0.0	0.000	0.0	0.532	1.6

^*a*^ The table summarizes within-run/day, between-run/day, and between-lot variability, including standard deviation and percent coefficient of variation (%CV) for CN values. Positivity rates with 95% confidence intervals are provided for each panel member at each site.

^*b*^ Excludes five unread cycles.

## DISCUSSION

Culture-based testing has long been utilized as the traditional method of laboratory evaluation of HSV; however, the more recent emergence of validated NAAT assays for detection and differentiation of HSV-1, HSV-2, and VZV is rapidly replacing culture as the gold standard for detection. PCR-based assays have been considered a preferred method for HSV and/or VZV detection due to their sensitivity, efficiency, and sample transport conditions compared to viral culture assays (12–14). While the advantages of PCR-based testing over viral culture are well established, it is also important to consider how the Alinity m HSV-1 & 2/VZV assay compares with currently available molecular diagnostic platforms. The assay provides multiplex detection and differentiation of HSV-1, HSV-2, and VZV from a single-lesion specimen, aligning with current trends toward syndromic and multiplex testing approaches. The Alinity m system offers a fully automated workflow integrating nucleic acid extraction, amplification, and detection, thereby minimizing hands-on time and reducing operator-dependent variability. In addition, the platform supports random-access and continuous loading, allowing prioritization of urgent specimens and improving laboratory workflow efficiency compared to batch-based systems.

HSV is an incurable infection, and appropriate use of diagnostic tests and prevention of transmission to sexual partners are key factors to consider for management of these infections (11). Differentiation of HSV-1 and HSV-2 is important to provide patients with information regarding expected prognosis of genital herpes (11). 2017 European guidelines for the management of genital herpes recommend HSV typing into HSV-1 and HSV-2 for all patients with first-episode genital herpes to guide counseling and management (4).

Detection of additional infectious agents such as *Treponema pallidum* from the same sample can further enhance the diagnostic capabilities of PCR-based assays (15).

Alinity m HSV 1 & 2/VZV assay is a multiplex assay that can detect and differentiate a panel of herpes viruses (HSV-1, HSV-2, and VZV) from the same sample. Analytical sensitivity in this study was determined using viral stocks quantified in TCID_50_/mL, which reflects viral infectivity rather than absolute genome copy number. The relationship between TCID_50_ and viral genome equivalents may vary depending on the viral strain, culture conditions, and phase of harvest. Therefore, LoD values expressed in TCID_50_/mL may not be directly comparable across analytes or with assays calibrated to genome copies. The positivity cutoffs (CN thresholds) for HSV-1, HSV-2, and VZV were predefined during assay development prior to initiation of the clinical study based on analytical and characterization studies and were not modified based on clinical specimen results in this evaluation. Given that the specimen collections were performed on symptomatic patients with lesions by trained clinicians, an endogenous cellular control was not required and not included in this assay to assess specimen adequacy; however, an internal control to monitor nucleic acid extraction and amplification efficiency was included for each sample.

The Alinity m HSV 1 & 2/VZV assay demonstrated high clinical sensitivity and specificity across all three viruses as expected for NAAT assays, indicating its reliability in detecting HSV-1, HSV-2, and VZV. The clinical sensitivity for the assay ranged from 95.7% to 98.4%, while specificity was between 99.3% and 100% across all three analytes. The discordant results where the specimens were positive by CC but negative by Alinity m assay were minimal. The competitive interference study shows that the presence of high concentrations of one analyte does not interfere with the detection of other analytes at lower concentrations, which is crucial for accurate detection in clinical samples that may contain multiple viral targets.

For HSV-1 and HSV-2, some specimens had CN values near the positivity cutoff, suggesting low-level target detection by the Alinity m assay, which was not observed with the comparator assays. The high clinical specificity of the Alinity m HSV 1 & 2/VZV assay reduces the likelihood of false-positive results, which is crucial for patient management and treatment decisions. The high clinical sensitivity ensures that most true positive cases are detected, minimizing the risk of missed diagnoses. Comparison of assay performance of the Alinity m HSV 1 & 2/VZV assay with two other PCR assays helps in understanding its relative efficacy. The assay’s ability to detect low-level targets that the comparator assays might miss could be a significant advantage in clinical settings. Use of a composite comparator (CC) method, where a specimen was considered positive if either comparator assay was positive, minimizes the potential for false-negative classification from an individual comparator assay and may introduce bias in classification of discordant results, especially for specimens with a low-level target. However, the testing of these specimens with a third comparator assay (e.g., third NAAT or sequencing) to confirm the results was not feasible due to limited sample volume. Since multiple specimens were obtained from some subjects, some degree of intra-subject correlation cannot be excluded and may influence performance estimates. The observed positivity rate reflects a broad, real-world population of patients with lesions suspected of HSV or VZV infection. Inclusion of both adult and pediatric populations, as well as variation in the lesion stage at the time of collection, may impact interpretability compared to more selectively defined cohorts. Although the assay reports a CN value for each positive sample, its primary utility lies in its robust qualitative performance. Collectively, these findings underscore the assay’s strong diagnostic capabilities and support its value as a reliable tool for accurate detection of HSV and VZV in clinical practice.

### Conclusions

The Alinity m HSV 1 & 2/VZV assay is a sensitive, specific, and reliable assay for detection of HSV and VZV from cutaneous and mucocutaneous lesion swab specimens. The assay has a low limit of detection of 5.90 TCID_50_/mL for HSV-1, 2.07 TCID_50_/mL for HSV-2, and 0.055 TCID_50_/mL for VZV. The 1,291 specimens tested showed a sensitivity range of 95.7% to 98.4% for HSV-1, HSV-2, and VZV. The specificity was >99% for all targets tested. This assay has demonstrated high clinical sensitivity and specificity for all three analytes.
